# The fourth scientific discovery paradigm for precision medicine and healthcare: Challenges ahead

**DOI:** 10.1093/pcmedi/pbab007

**Published:** 2021-04-16

**Authors:** Li Shen, Jinwei Bai, Jiao Wang, Bairong Shen

**Affiliations:** Institutes for Systems Genetics, Frontiers Science Center for Disease-related Molecular Network, West China Hospital, Sichuan University, Chengdu 610041, China; Library of West-China Hospital, Sichuan University, Chengdu 610041, China; Institutes for Systems Genetics, Frontiers Science Center for Disease-related Molecular Network, West China Hospital, Sichuan University, Chengdu 610041, China; Institutes for Systems Genetics, Frontiers Science Center for Disease-related Molecular Network, West China Hospital, Sichuan University, Chengdu 610041, China

**Keywords:** data-intensive scientific discovery, the fourth paradigm, biomedical data diversity, precision medicine and healthcare

## Abstract

With the progression of modern information techniques, such as next generation sequencing (NGS), Internet of Everything (IoE) based smart sensors, and artificial intelligence algorithms, data-intensive research and applications are emerging as the fourth paradigm for scientific discovery. However, we face many challenges to practical application of this paradigm. In this article, 10 challenges to data-intensive discovery and applications in precision medicine and healthcare are summarized and the future perspectives on next generation medicine are discussed.

## Introduction

The scientific discovery paradigm (SDP) provides a mature and routine framework for asking scientific questions, developing methods or strategies to answer such questions, and also includes ways to explain the experimental results or the observed data. In the last two decades, the SDP in life sciences has shifted fast, especially with progression of the human genome project. A paradigm shift, sometimes also called a 'scientific revolution', occurs when the existing paradigm cannot efficiently solve the challenges faced and a new paradigm is needed to deal with the challenges. For example, in bioinformatics, a small paradigm shift we refer to here as the bioinformatics scientific research model (SRM), emerged with accumulation of DNA sequencing data. Since then, a batch of new genes has been discovered by pattern identification with models trained using known gene structure patterns. Well-known bioinformatics tools and databases including CLUSTAL W,^1^ MEGA,^2^ PDB^3^*etc*., were developed within the bioinformatics SRM. Traditional experimental paradigms can only discover new genes one by one through time-consuming and labor-intensive methods. Complex biological systems, however, often function by interactions between many genes, proteins, or other components via pathways, modules, or networks. Bioinformatics has contributed to acceleration in life sciences by fast, efficient, high throughput, and computational methods, enabling investigation of biological and medical problems at systemic levels. The microarray, yeast two-hybrid assay, and evolutionary modeling promoted the paradigm shifting to systems biology, which aimed to reconstruct the interaction or synergistic network to explain emergence properties in a system. Systems biological tools such as gene ontology,^4^ KEGG^5^ and Cytoscape,^6^*etc*., were then developed and widely used. But for clinical translation, genome functional discoveries cannot be applied directly to treatment of patients because of heterogeneities among diseases and patients. Cell-line or animal-model based biological findings need to be validated with patient samples before clinical applications. Therefore, translational and precision medicine SRMs have been proposed to integrate genotypic and phenotypic information for personalized prediction and treatment of diseases.^7, 8^

Although paradigms in life sciences have shifted frequently in the past 20 years, data accumulation is always the driving force for scientific revolution. In the future, data will remain one of the most essential parts for successful scientific paradigm shifts; however, the quality, quantity, and diversity of biomedical data will pose key challenges for our future precision medicine and healthcare.

## The fourth paradigm: data-intensive scientific discovery

**Figure 1.**
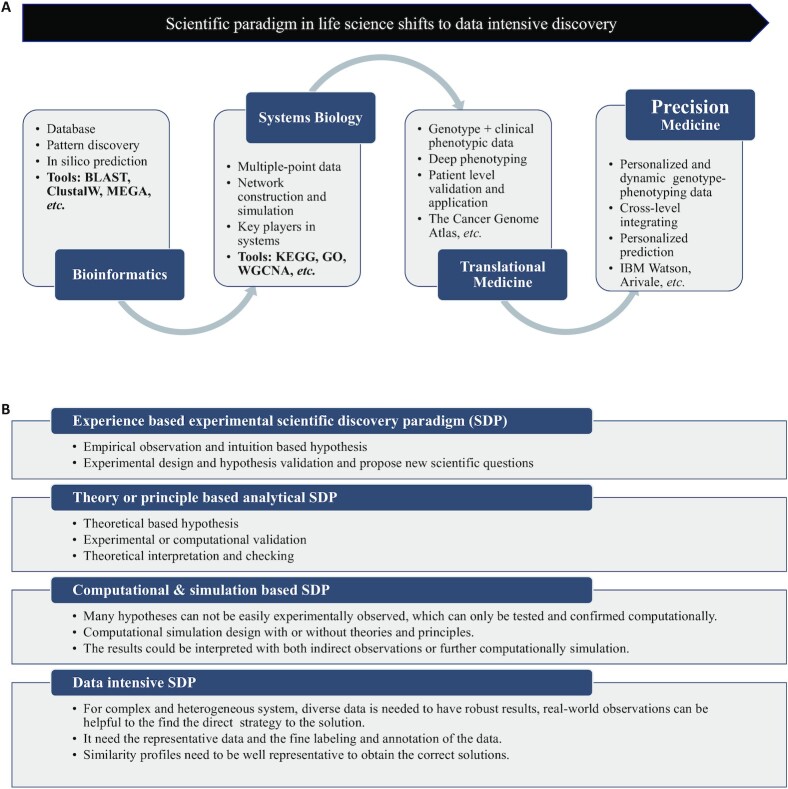
(A) Scientific paradigm shifts in last two decades. (B) Four scientific discovery paradigms.

As shown in Fig. 1A, paradigm shifts in the life sciences over the last two decades present a very salient characteristic, i.e. more and more data are needed for scientific discovery in life sciences. The bioinformatics SRM emerged with progression of the human genome project. As more DNA sequencing data were accumulated, gene structures in the genome could be compared and the DNA string patterns specific to protein coding genes, non-coding RNAs, and the regulatory elements therefore could be identified for prediction of new genes. Since then, many databases have been established for investigations of biological questions. Compared with traditional biostatistics discipline, which can do nothing with a single DNA or protein sequence, bioinformatics tools provide researchers with enormous DNA information resources for ortholog or paralog screening, phylogenetic tree construction, 3D structure modeling, functional specificity estimation, and so on.^9–12^ For the systems biology SRM, the first step was reconstruction of the biological network by top-down or bottom-up strategies, where multiple points or correlated data are demanded to infer the interactions between nodes and the structures of networks or systems.^13–15^ The translational medicine and precision medicine SRMs further need clinical and personalized data for deep phenotyping and personalized diagnosis and treatment of patients.^16–18^

Figure 1B displays the four SDPs, with traditional SDPs including experimental, theoretical, and computational ones. Compared with the three traditional paradigms, the fourth paradigm, i.e. data-intensive SDP, has emerged in modern technologies, including high throughput sequencing, cloud computing, smart sensors, digital medicine, Internet of Everything, next generation artificial intelligence, and so on.^19,20^ Especially for complex and heterogeneous systems such as ecological systems, cancer, and many chronic diseases, it is difficult to describe and understand such complexity using simple rules or theories. The fourth paradigm will be an important complementary solution to the other three paradigms. Complex and chronic diseases are often caused by interactions of many factors such as genetic events, lifestyles, and environmental factors. The fourth paradigm provides a way to deal with personalized diagnosis and treatment with huge amounts of patient information by calculating similarities between the query patients and profiles in the targeted databases. However, the prerequisite for the success of this paradigm is that the data accumulated are 'big' enough to cover all possibilities. Furthermore, the science based on this big data needs new algorithms for discoveries of new rules, principles, key players, and mechanisms for the understanding and controlling of the life systems.

## Biomedical data diversity and standardization

As the fourth paradigm for scientific research is characterized by the intensity of data, high quality data accumulation will be an essential step for data-driven personalized and precision medicine. Biomedical data such as data at molecular, cellular, tissue, individual, and population levels, are usually diverse and heterogeneous. These could be basic scientific data from laboratories, or real world clinical or health status data, and the data could be dynamic, evolutionary, and spatiotemporal. The following three challenges will be faced in application of the fourth paradigm to precision medicine practice.

### Challenge 1: Data standardization for communication

To collect big biomedical data, the data formats, terminologies, and relationships should be standardized.^21^ Clinical scientists need to share their data and information, especially for rare disease description, to improve the diversity and representativeness of the collected data. The biomedical data are huge considering the disease types, personalized genetics, dynamic lifestyles, environmental factors, as well as the synonyms and complex relationships.

### Challenge 2: Data sharing and privacy preservation

It has been reported that several genes could be combined with known personal information to re-identify personal features, such as 3D facial reconstruction, inference of voices, and family names. To protect personal privacy, the clinical information need to be desensitized, or perhaps even 'noise' introduced to the data via differential privacy to secure multi-party computation and sharing.

### Challenge 3: Data measurability and spatiotemporal signals

Spatiotemporal molecular medicine is becoming the new discipline for investigation of dynamic and evolutionary human health status. Although modern smart sensor technologies can be applied to collection of physiological information and molecular level information, it is not easy to collect dynamic signals and data. Investigations such as gene expression and microbiota ecological dynamics are still very challenging.

## The explainable model and actionable key data

To understand the complexity and identify specific patterns hidden in the big data, personalized models are necessary. Data-intensive algorithms, such as deep learning models, need to be explainable and actionable for precision medicine and healthcare.^22^

### Challenge 4: Phenotype plasticity and model robustness

The health or disease status is determined by complex interactions of many genetic, lifestyle, and environmental factors.^23^ One disease phenotype could be associated with many genotypic factors, and the methods or solutions to transfer the disease to health status are not unique. The model constructions are not the same as traditional one-to-one mode but should be one-to-N mode, considering the plasticity of phenotype.

### Challenge 5: Explainable artificial intelligence (XAI) and precision medicine practice

Most traditional machine learning (ML) algorithms are models for classification, somewhat 'black boxes' in that their mechanisms and explanations remain unknown. It is difficult to apply these ML models to design of personalized treatment. XAI will be helpful to 'open the black box', facilitating trust of patients or clinical doctors in use of AI predictions in precision medicine practice.

### Challenge 6: Clinical observation and real world data-driven scientific discovery

For precision medicine and healthcare, clinical observation/questions and real world data are the two main resources in hospitals, which cannot be obtained from laboratories. There remains a challenge to propose good clinical questions for scientific investigation as these require insights and experiences from both clinical and basic science.

### Challenge 7: Experimental or computational verifiability

Discoveries of biomarkers, drug targets, and other key players based on the fourth paradigm need to be verified and validated with experiments (including clinical practice) or computational-aided simulations.^24^ These could require further improvement and re-validation before they can be safely and widely applied to medicine and healthcare practice.

### Challenge 8: From data and knowledge to general principles

Although an XAI-based model can explain an observation, further exploration in data-intensive research will be required to discover the general principles underlying the observed patterns. The principles can then be used to guide design of the strategies for better treatment of diseases.^25^ The fourth SDP is a complement to the other three paradigms and these can be integrated with each other to accelerate discovery in medicine.

## Translational application and cross-disciplinary education

Even if all scientific discoveries are aimed at applications, we are still short of qualified persons for the fourth SDP practice. The last two challenges concern application and education.

### Challenge 9: Smart application of data-intensive SDP to healthcare

As we have limited medical resources to combat widespread chronic diseases, data-intensive scientific discovery could be transferred to smart patient self-administration, especially chronic disease monitoring and controlling. Knowledge-guided chatbots could offer a way to improve the quality of diagnosis, outpatient consultation, and referral as well as treatment.^26^

### Challenge 10: Education and training for data-intensive SDP

To overcome the nine challenges in the life sciences as stated above, we need well-educated and trained clinicians and scientists. The next generation of medical doctors, researchers, and even patients, should be equipped with knowledge on data standardization, data security, knowledgebases, algorithms and models, *etc*., for cross-disciplinary studies using data-intensive SDP.

## Conclusions and future perspectives

The first three SDPs have been applied in most scientific fields, including physics, chemistry, engineering, *etc*. The fourth SDP is emerging and will evolve with big data science and technology. Data diversity and heterogeneity remain two main challenges in the life sciences. Disease profiles and data spaces for biomedical data are very big and still expanding with evolution of interactions between genetics, lifestyles, and environments.

Two well-known efforts, IBM Watson and Arivale's wellness project,^27,28^ have reported failures in healthcare, the main reason being that the data collected for their artificial intelligent modeling or analytics are not representative when faced with complex and personalized application. The healthcare industries need more well-labeled data, knowledge-guided models,^29^ and experienced human resources. With integration of the four paradigms, the challenges for the new paradigm applications, on the other hand, are also the opportunities for efforts to develop ontologies for standardization of data, to build knowledge databases for explainable artificial intelligence modeling, and to dig into the genotyping-phenotyping relationship for precision applications for precision medicine and healthcare practice.
